# Vitamin D can ameliorate premature ovarian failure by inhibiting neutrophil extracellular traps: A review

**DOI:** 10.1097/MD.0000000000033417

**Published:** 2023-03-31

**Authors:** Menglu Chen, Lailai Li, Yihui Chai, Yuqi Yang, Sibu Ma, Xiang Pu, Yunzhi Chen

**Affiliations:** a Guizhou University of Traditional Chinese Medicine, Guiyang City, Guizhou Province, China.

**Keywords:** immune response, inflammation, neutrophil extracellular traps, oxidative stress, premature ovarian failure, tissue fibrosis, vitamin D

## Abstract

The etiology of premature ovarian failure (POF) is mainly related to inflammatory diseases, autoimmune diseases, and tumor radiotherapy and chemotherapy; however, its specific pathogenesis has not been clarified. Vitamin D (VD), a fat-soluble vitamin, is an essential steroid hormone in the human body. Neutrophil extracellular traps (NETs) are meshwork structures that are formed when neutrophils are stimulated by inflammation and other factors and are closely associated with autoimmune and inflammatory diseases. Notably, VD inhibits NET formation and intervenes in the development of POF in terms of inflammatory and immune responses, oxidative stress, and tissue fibrosis. Therefore, this study aimed to theorize the relationship between NETs, VD, and POF and provide new ideas and targets for the pathogenesis and clinical treatment of POF.

**Brief summary**

VD may play an important role in POF by inhibiting NET formation, including inflammatory responses, immune responses, oxidative stress, and tissue fibrosis.

## 1. Introduction

Premature ovarian failure (POF) is a disease of the female reproductive system characterized by a decrease in the development of mature follicles in the ovaries and an increase in atretic follicles as the main pathological change.^[1]^ The morbidity of POF is increasing in young women as people lifestyles and dietary structures change. According to relevant data, approximately 45 million women in China have been diagnosed with POF, and the prevalence rate has reached 1/10,000, 1/1000, and 1/100 in women aged 20 seconds, 30 seconds, and 40 seconds, respectively.^[2]^ The disease not only seriously affects women physical and mental health but also imposes a huge burden on families and society.^[3]^ Currently, estrogen and progestin replacement, ovulation promotion, and immunotherapy are commonly used to treat POF, but they cannot alter the pathological nature of reduced mature follicle development.

**Figure 1.**
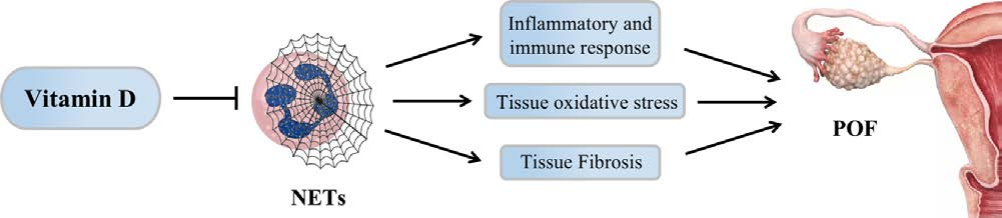
Regulation mechanism of vitamin D on POF through inhibition of NETs. NETs = neutrophil extracellular trap network, POF = premature ovarian failure.

The pathogenesis of POF is unclear, but the most recent research demonstrates that tissue fibrosis, oxidative stress, immunological response, and inflammation are significant contributors to the pathogenesis of POF, while excessive deposition of neutrophil extracellular traps (NETs) can promote the release of cytokines, which can harm tissue function by causing inflammation, immunology, oxidative stress, and fibrosis. Interestingly, investigations have discovered that VD can inhibit the formation of NETs in tissues, and can promote follicle development and maturation by regulating oxidative stress and steroid production pathways in the ovarian granulosa cells. Therefore, we propose the scientific hypothesis that VD may prevent POF by inhibiting NET formation and further explore its potential mechanism, with the aim of providing new ideas for the clinical treatment of POF (Fig. 1).

## 2. Influence of inflammatory response, autoimmunity, oxidative stress, and tissue fibrosis on the pathological changes of POF

Current research suggests that the pathogenesis of POF may be related to the inflammatory response, autoimmunity, oxidative stress, and tissue fibrosis caused by radiotherapy and chemotherapy.^[4,5]^

### 2.1. Inflammation and immune response to POF

**Figure 2.**
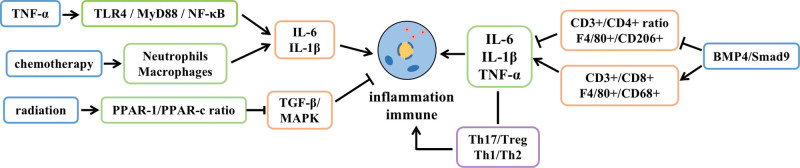
Inflammation and immune response to POF. POF = premature ovarian failure.

Inflammation and autoimmune responses can lead to tissue damage and dysfunction. It has been shown that tumor necrosis factor α (TNF-α) binding to its receptor in the ovary could induce elevated levels of nuclear factor kappa-B (NF-κB), activate the TLR4/MyD88/NF-κB signaling pathway, induce the secretion of various pro-inflammatory cytokines, and result in an inflammatory response in the ovary.^[6,7]^ Chemotherapy causes a large number of neutrophils and macrophages to enter the ovarian tissue, which are distributed mainly in the corpus luteum and atretic follicles, and promotes increased levels of pro-inflammatory factors IL-6, Interleukin-1β (IL-1β), and TNF-α, ultimately inducing an inflammatory response in the ovarian tissue.^[8]^ In addition, radiation can induce ovarian hypofunction by upregulating the ratio of PPAR-1 mRNA to PPAR-c mRNA in oocytes and ovarian granulosa cells, as well as by downregulating the TGF-β/MAPK signaling pathway and promoting inflammatory responses.^[9]^ Meanwhile, high-fat and high-sugar diets can stimulate the secretion of ovarian pro-inflammatory cytokines IL-1β, IL-6, and TNF-α by inhibiting the BMP4/Smad9 signaling pathway, decreasing the ratio of helper T cells (CD3+/CD4+) and the ratio of M2 type macrophages (F4/80+/CD206+), and increasing the ratio of activated T cells (CD3+/CD8+) and the ratio of M1 type macrophages (F4/80+/CD68+), causing an imbalance in immune regulation and induction of an inflammatory response in ovarian tissues.^[10]^ Pro-inflammatory cytokines can also induce an imbalance in the ratio of Helper T-cell 17 (Th17)/regulatory cells and helper T-cell 1 (Th1)/helper T-cell 2 in the ovary and activate monocytes to regulate the immune response, leading to follicular atresia and ovarian failure^[11,12]^ (Fig. 2).

### 2.2. Effect of oxidative stress on POF

**Figure 3.**
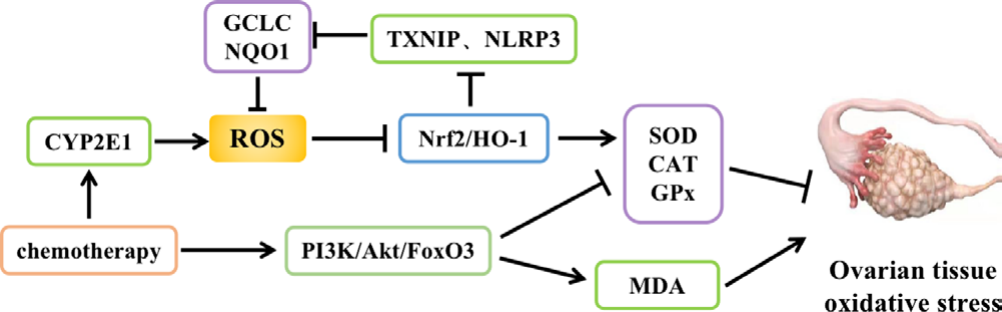
Effect of oxidative stress on POF. CAT = catalase, GPx = glutathione peroxidase, MDA = malondialdehyde, POF = premature ovarian failure, ROS = reactive oxygen species, SOD = superoxide dismutase.

Reactive oxygen species (ROS) are byproducts of normal body metabolism and are beneficial to cells and tissues at physiological levels, whereas excess ROS can promote oxidative stress and induce cell apoptosis.^[13]^ Chemotherapeutic drugs can promote the release of ROS through CYP2E1, inhibit the production of the antioxidant nuclear factor NF-E2-related factor (Nrf2) and heme oxygenase 1 (HO-1), and downregulate the Nrf2/HO-1 signaling pathway, which induces DNA double-strand breaks and oxidative bases in ovarian granulosa cells and decreases the activity of antioxidant enzymes such as superoxide dismutase (SOD), catalase (CAT), and glutathione peroxidase (GPx) in tissues, causing oxidative stress in ovarian tissues.^[14]^ Meanwhile, downregulation of Nrf2 also leads to the activation of thioredoxin-interacting protein and NLR family pyrin domain containing 3 inflammatory vesicles, which induce decreased expression of antioxidant enzymes such as glutamate-cysteine ligase catalytic subunit, HO-1, and NAD(P)H quinone dehydrogenase 1, resulting in a significant increase in oxidative stress levels and excessive accumulation of ROS in the ovary, impairing its physiological function.^[15]^ In addition, chemotherapy can lead to a significant increase in ovarian oxidative stress levels and induce ovarian hypofunction by stimulating the PI3K/Akt/FoxO3a signaling pathway, downregulating the expression levels of SOD and glutathione, and upregulating the expression levels of the pro-oxidant malondialdehyde (MDA) in the ovary^[16]^ (Fig. 3).

### 2.3. Effect of tissue fibrosis on POF

**Figure 4.**
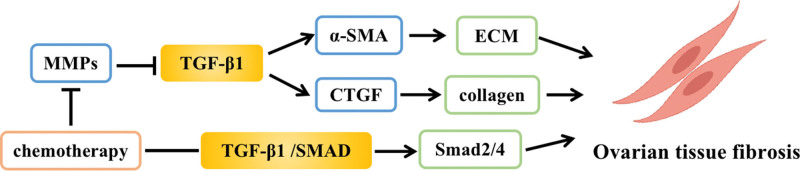
Effect of tissue fibrosis on POF. CTGF = connective tissue growth factor, ECM = extracellular matrix, MMPs = matrix metalloproteinases, POF = premature ovarian failure.

The accumulation of extracellular matrix (ECM) and sustained activation of fibroblast production are the main pathogenic mechanisms of tissue fibrosis.^[17]^ Transforming growth factor β1 (TGF-β1), an important regulator of fibroblast phenotype and function, promotes the synthesis and deposition of ECM and contributes to the progression of tissue fibrosis.^[18]^ Chemotherapeutic drugs can upregulate TGF-β1 expression in the ovary by reducing the levels of matrix metalloproteinases (MMPs), which have antifibrotic activity, activate α-smooth muscle actin (α-SMA), inhibit ECM degradation, recruit fibroblast accumulation, and lead to ovarian tissue fibrosis.^[19]^ In addition, chemotherapeutic agents can induce increased compliance of ovarian tissue Smad2/4 expression by modulating the TGF-β/SMAD axis, leading to tissue fibrosis and ovarian functional impairment.^[20]^ As an important factor in the development of ovarian tissue fibrosis, connective tissue growth factor (CTGF) interacts with TGF-β1 to regulate the development and progression of tissue fibrosis. Overexpression of TGF-β1 induced CTGF to promote increased collagen production through a paracrine mechanism and accelerated the development of ovarian tissue fibrosis^[19]^ (Fig. 4).

## 3. VD interfere with the pathological process of POF by inhibiting tissue inflammation and immune response, oxidative stress, and tissue fibrosis process

Vitamin D (VD) is a fat-soluble steroid hormone. The 7-dehydrocholesterol in the skin is irradiated by UV light to produce VD precursors, which are hydroxylated by 27-hydroxylase in the liver to form 25-hydroxy vitamin D_3_ [25(OH)D_3_] and then hydroxylated by 1α-hydroxylase in the kidney tubules to form biologically active 1,25-dihydroxy vitamin D_3_ [1,25(OH)_2_D_3_], which binds to the vitamin D receptor (VDR) in tissues to exert its biological effects and is finally inactivated by 24-hydroxylase and excreted from the body. Recent studies have reported that VD exerts a protective effect on body tissues by inhibiting tissue inflammation, immune response, oxidative stress, and fibrosis progression.

### 3.1. VD inhibit inflammation and immune response

**Figure 5.**
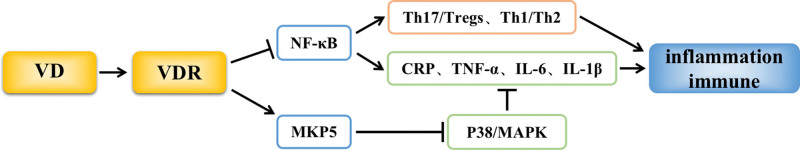
VD inhibit inflammation and immune response. CRP = C-reactive protein, VD = vitamin D, VDR = vitamin D receptor.

VD, an important factor that regulates immune cell function in vitro and in vivo, plays an important role in the antiinflammatory and antiimmune responses. Studies have shown that VDR is a key regulator of innate immune cell differentiation.^[21]^ Following binding to VDR, 1,25(OH)_2_D_3_ activates macrophage antiinflammatory function, antagonizes the activation of NF-κB signaling in T cells, reduces Th1 and Th17 cell production, and upregulates the ratio of Helper T-cell 2 and regulatory cells in tissues.^[22–24]^ VDR can be expressed in dendritic cells, CD4+, CD8 + T cells, and other immune cells, and bind to 1,25(OH)_2_D_3_ to maintain a healthy immune system.^[25]^ VD also slows down the inflammatory response of the body and exerts a suppressive effect on the adaptive immune system by reducing immunoglobulin E expression, inhibiting IκB kinase activity, regulating the NF-κB signaling pathway to upregulate the levels of antiinflammatory cytokines, and downregulating the levels of pro-inflammatory cytokines.^[26]^ As a stress-activated kinase, P38 mitogen-activated protein kinase increases pro-inflammatory cytokine production and induces inflammatory responses. 1,25(OH)_2_D_3_ can upregulate tissue-dephosphorylated MAPK phosphatase 5 levels and inhibit P38 mitogen-activated protein kinase activation to reduce inflammatory cytokine production, thereby decreasing ovarian inflammatory responses.^[23]^ In addition, 1,25(OH)_2_D_3_ binding to VDR in tissues directly inhibits NF-κB binding to the COX-2 promoter, reduces prostaglandin synthesis, decreases the expression of pro-inflammatory factors, such as C-reactive protein, TNF-α, IL-6, and IL-1β, and ultimately ameliorates the inflammatory response and tissue damage^[27]^ (Fig. 5).

### 3.2. VD suppress oxidative stress

**Figure 6.**
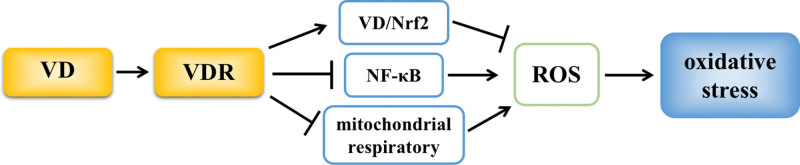
VD inhibit oxidative stress. VD = vitamin D.

VD, a steroid hormone with good antioxidant activity, can reverse high-fat diet-induced oxidative stress by elevating SOD and GPX and reducing MDA concentrations in tissues.^[28]^ VD can also activate the VD/ Nrf2 signaling pathway in tissues and upregulate the expression of antioxidant factors, such as SOD, CAT, and GPx, to reduce the level of ROS.^[29]^ Additionally, VDR is an important regulator of cellular mitochondrial respiratory activity. Activated VDR can significantly inhibit cellular mitochondrial respiratory activity, reduce ROS release, and protect tissues from oxidative stress damage.^[30]^ Meanwhile, it has been shown that VD can effectively reduce total oxidative status, oxidative stress index and MDA levels and increase total antioxidant response and total thiol levels in tissues by inhibiting the activation of NF-κB signaling pathway in tissues, thereby alleviating tissue damage caused by oxidative stress.^[31]^ In addition, VD can decrease the levels of MDA and total oxidative status, increase the total antioxidant status level in the ovaries, reduce ovarian follicle degeneration and matrix degeneration, and eventually improve ovarian oxidative damage caused by hyperthyroidism^[32]^ (Fig. 6).

### 3.3. VD inhibit tissue fibrosis

**Figure 7.**
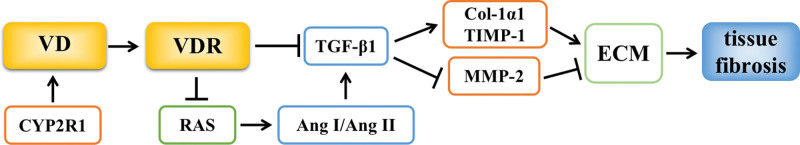
VD inhibit tissue fibrosis. RAS = renin-angiotensin system, VD = vitamin D.

The TGF-β signaling pathway has been shown to be one of the major pathways that promote ECM accumulation and exacerbate tissue fibrosis.^[33]^ VDR, as a negative regulator of fibroblast activation, antagonizes the pro-fibrotic effects of TGF-β1 and has efficient antifibrotic activity, and 1,25(OH)_2_D_3_ significantly upregulates VDR levels in tissues, inhibits the pro-fibrotic activity of TGF-β1/α-SMA signaling pathway, and reduces the stimulatory effect of TGF-β1 on fibroblasts and ECM deposition in tissues, thus improving tissue fibrosis.^[34]^ In addition, it was shown that deficiency of CYP2R1, an important hydroxylase of VD, can lead to tissue fibrosis, while supplementation with VD can upregulate the expression level of CYP2R1 in tissues, inhibit the TGF-β1 signaling pathway, reduce the expression of pro-fibrotic genes collagen type I α 1 and tissue inhibitor of metalloproteinase-1, and elevate the expression of the antifibrotic gene MMPs-2, thus alleviating the development of tissue fibrosis.^[35]^ Studies have shown that the renin-angiotensin system is one of the important mediators of tissue fibrosis induction, which can regulate the conversion of angiotensin I to angiotensin II and upregulate tissue TGF-β1 expression to induce fibroblast proliferation; in 1,25(OH)_2_D_3_ can bind to VDR in tissues and prevent tissue fibrosis formation by inhibiting renin-angiotensin system activation and reducing fibrogenic factor and ECM formation^[34]^ (Fig. 7).

Experimental studies have shown that VD can promote follicle development and maturation by regulating oxidative stress and steroid production pathways in the ovarian granulosa cells.^[36,37]^ VD plays an important role in the antiinflammatory, immunosuppressive, antioxidant, and antitissue fibrosis aspects of the body. Therefore, VD may interfere with the pathogenesis of POF by suppressing inflammation, immune response, oxidative stress, and tissue fibrosis to improve POF.

## 4. NETs involved in the pathological process of POF by promoting tissue inflammation and immune response, oxidative stress, as well as tissue fibrosis processes

NETs are extracellular fibers consisting mainly of a meshwork of extracellular DNA, nucleoproteins, and serine proteases released by neutrophils.^[38]^ Upon initiation of NETosis, neutrophil elastase (NE) and myeloperoxidase degrade the cytoskeleton. Histones are disassembled by peptidylarginine deiminase 4, which allows genomic DNA modified by peptides and proteins to fill the cytoplasm. It is released outside the cell when DNA expansion pressure breaks the cell membrane. At this point, NE, myeloperoxidase, histones, and other proteases are embedded in the DNA backbone, eventually forming NETs outside the neutrophil and becoming a part of the body innate immune system.^[39,40]^ However, excessive deposition of NETs can activate multiple signaling pathways and stimulate cytokine production, leading to inflammatory and immune responses, oxidative stress, and tissue fibrosis.^[41]^

### 4.1. NETs promote inflammation as well as immune response

**Figure 8.**
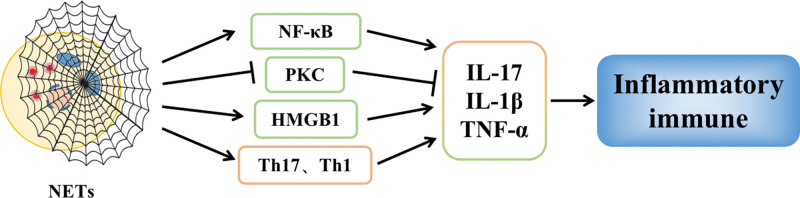
NETs promote inflammation and immune response. HMGB1 = high-mobility group box 1, NETs = neutrophil extracellular trap network, PKC = protein kinase C.

NETs promote the development of inflammatory responses and are also a source of autoantigens, which are widely involved in the development and progression of inflammatory and autoimmune diseases in the body. NETs activate tissue macrophages and elicit antigen-specific T cell responses, producing pro-inflammatory Th17 and Th1 cells and secreting pro-inflammatory cytokines such as interleukin 17, IL-1β, and TNF-α to induce tissue inflammatory responses and adaptive immune responses.^[40,42]^ In addition, miRNAs from NETs can act on macrophages to downregulate protein kinase C levels and induce excessive TNF-α production in tissues.^[43]^ In parallel, NETs promote the interaction between NF-κB regulators and IκB kinase to activate the NF-κB signaling pathway, release large amounts of pro-inflammatory cytokines, and induce inflammatory and immune responses in tissues.^[44]^ In addition, adenylate activated protein kinase (AMPK), a sensor that regulates cellular energy metabolism, has powerful antiinflammatory effects; while excessive release of NETs inhibits AMPK activity and depresses phagocytosis of apoptotic cells by macrophages, as well as exacerbates tissue inflammatory responses by activating the release of the pro-inflammatory mediator high-mobility group box 1, which promotes the secretion of pro-inflammatory factors^[45]^ (Fig. 8).

### 4.2. NETs promote oxidative stress

**Figure 9.**
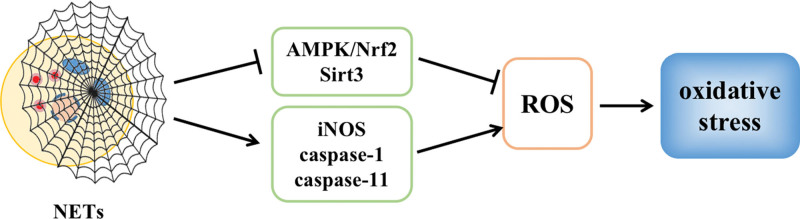
NETs promote oxidative stress. iNOS = inducible nitric oxide synthase, NETs = neutrophil extracellular trap network.

NET formation is an important factor in the occurrence of oxidative stress in tissues. When injury and inflammation occur, the body releases chemokines, induces neutrophil recruitment, produces large amounts of ROS, and induces the formation of NETs through the reduced coenzyme II oxidase-ROS-dependent pathway while reducing Nrf2 production, inhibiting the AMPK/Nrf2 signaling pathway, depressing SOD and CAT activity in tissues, and inducing the development of oxidative stress in tissues.^[46,47]^ It has been shown that nanoalumina can induce excessive formation of NETs, further inhibit the activities of SOD, CAT, and glutathione, upregulate the expression levels of inducible nitric oxide synthase, caspase-1, and caspase-11, and exacerbate tissue oxidative stress damage.^[48]^ Sirtuin 3 has been shown to be a mitochondria-dependent deacetylase that protects tissues from oxidative stress damage. In contrast, excessive NET formation can induce NET-mediated oxidative stress damage in tissues by inhibiting Sirtuin 3 activity and reducing SOD2 transcription in tissues^[49]^ (Fig. 9).

### 4.3. NETs induce tissue fibrosis

**Figure 10.**
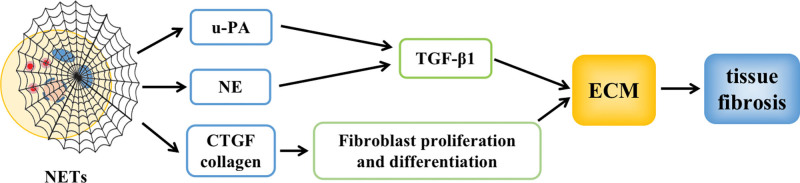
NETs promote oxidative stress. CTGE = connective tissue growth factor, NE = neutrophil elastase, NETs = neutrophil extracellular trap network.

NETs can promote fibrotic processes in various tissues, such as the liver and skeletal muscles.^[50,51]^ When tissue is injured, a large number of neutrophils are recruited to the site of injury, inducing NETosis, which produces NETs that stimulate cells to create urokinase-type plasminogen activator, release TGF-β1 enclosed in the ECM, activate the TGF-β1/α-SMA signaling pathway, and downregulate the level of MMPs, promoting collagen production and ECM deposition in tissues, leading to tissue fibrosis.^[52]^ NE is an important component of NETs. Studies have shown that NE not only activates TGF-β and induces ECM deposition but also directly enters fibroblasts, promoting their proliferation and differentiation capacity, ultimately leading to tissue fibrosis.^[53]^ In addition, NETs can also promote fibroblast proliferation and migration and induce the conversion of fibroblasts to myofibroblast phenotype by upregulating the expression of CTGF, promoting collagen production, and ultimately aggravating tissue fibrosis^[54]^ (Fig. 10).

The development of POF is dependent on the involvement of inflammation and immune response, oxidative stress, and tissue fibrosis, whereas the excessive formation of NETs may exacerbate inflammation and immune response, oxidative stress, and fibrosis in the organism. Therefore, we speculated that excessive NET formation may drive the pathological development of POF.

## 5. VD may prevent POF by inhibiting the formation of NETs

Long-term administration of low-dose VD has been reported to significantly inhibit NET formation in tissues, which in turn upregulates vascular endothelial growth factor expression and reduces the production of pro-inflammatory cytokines, thereby improving tissue microvascular growth and alleviating the inflammatory response.^[55]^ Therefore, we hypothesized that VD may improve POF by inhibiting NET formation and attenuating inflammation, immune response, oxidative stress, and fibrosis in ovarian tissues.

Follicular atresia is the main pathological change in POF and is closely related to inflammatory responses and autoimmune abnormalities.^[56]^ Both VD and NETs regulate the NF-κB signaling pathway and antigen-specific T cell responses. Thus, it is hypothesized that POF patients may be treated with appropriate VD supplementation to inhibit NET formation, improve the tolerance of ovarian tissue to autoimmunity, and alleviate ovarian inflammation and immune response.

Excessive ROS production in the body has been shown to be associated with inhibition of follicular development and damage to ovarian granulosa cells in mammals. NETs inhibit Nrf2 production and induce tissue oxidative stress, whereas VD regulates cellular mitochondrial respiratory activity and reduces tissue ROS levels. Therefore, VD may regulate the VD/Nrf2 signaling pathway by binding to VDR in the ovarian epithelium, inhibiting the formation of NETs, reducing ROS production, and upregulating the level of antioxidant enzymes to alleviate oxidative stress-induced decline in ovarian function.

**Figure 11.**
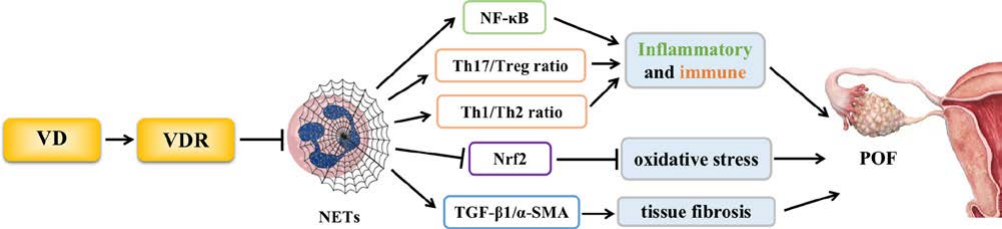
VD may interfere with POF by inhibiting the production of NETs, to modulate the said pathways and targets, suppressing ovarian tissue inflammation and immune responses, oxidative stress, as well as tissue fibrosis. NETs = neutrophil extracellular trap network, POF = premature ovarian failure, VD = vitamin D.

Furthermore, TGF-β1 promotes ECM deposition and ovarian fibrosis by activating the fibrosis-related gene α-SMA in ovarian tissues. NETs promote tissue TGF-β1 production, whereas VD downregulates the TGF-β1/α-SMA pathway to inhibit tissue fibrosis. Therefore, we propose the hypothesis that VD may reduce ECM deposition in ovarian tissue by inhibiting the formation of NETs and downregulating the TGF-β1/α-SMA signaling pathway, thereby alleviating ovarian fibrosis and improving ovarian function (Fig. 11).

## 6. Limitation and suggestion

However, the pathogenesis of POF remains unclear. Therefore, it is one of the most important hotspots for further investigation of new targets for the treatment of POF and the development of highly effective drugs with no significant adverse reactions for the prevention and treatment of POF. NETs have become key targets for the development of pathological changes and clinical treatment of various diseases and are closely related to various inflammatory and autoimmune diseases. VD may interfere with POF by regulating ovarian granulosa cell apoptosis and tissue inflammatory responses. VD may play an important role in POF by inhibiting the formation of NETs, including inflammatory response, immune response, oxidative stress, and tissue fibrosis; however, the exact mechanism is still unclear. In the future, we can carry out high-quality research in vitro, in vivo, and in clinical treatment, hoping to provide new directions and new ideas for the clinical treatment of POF, and to provide a theoretical basis and scientific support for the development of emerging targeted drugs for POF.

## Acknowledgments

We thank Yihui Chai and Yuqi Yang for assistance in data collection and organization and Sibu Ma for his help with data analysis.

## Author contributions

**Conceptualization:** Menglu Chen, Lailai Li, Yunzhi Chen.

**Data curation:** Menglu Chen, Yihui Chai, Yuqi Yang, Sibu Ma.

**Funding acquisition:** Lailai Li.

**Validation:** Xiang Pu.

**Visualization:** Yihui Chai, Yuqi Yang.

**Writing – original draft:** Menglu Chen.

**Writing – review & editing:** Lailai Li, Xiang Pu, Yunzhi Chen.

## References

[1] Igboeli P El Andaloussi A Sheikh U. Intraovarian injection of autologous human mesenchymal stem cells increases estrogen production and reduces menopausal symptoms in women with premature ovarian failure: two case reports and a review of the literature. J Med Case Rep. 2020;14:108. 32680541 10.1186/s13256-020-02426-5 PMC7368722

[2] Abdelzaher WY Abdel-Hafez SMN Rofaeil RR. The protective effect of fenofibrate, triptorelin, and their combination against premature ovarian failure in rats. Naunyn Schmiedebergs Arch Pharmacol. 2021;394:137–49. 32924068 10.1007/s00210-020-01975-2

[3] Melekoglu R Ciftci O Eraslan S. Beneficial effects of curcumin and capsaicin on cyclophosphamide-induced premature ovarian failure in a rat model. J Ovarian Res. 2018;11:33. 29699594 10.1186/s13048-018-0409-9 PMC5918567

[4] Chen H Xiao L Li J. Adjuvant gonadotropin-releasing hormone analogues for the prevention of chemotherapy-induced premature ovarian failure in premenopausal women. Cochrane Database Syst Rev. 2019;3:Cd008018. 30827035 10.1002/14651858.CD008018.pub3 PMC6397718

[5] Li D Chen Y Qi L. Differentially expressed genes in cisplatin-induced premature ovarian failure in rats. Anim Reprod Sci. 2013;137:205–13. 23266200 10.1016/j.anireprosci.2012.11.011

[6] Ibrahim MA Albahlol IA Wani FA. Resveratrol protects against cisplatin-induced ovarian and uterine toxicity in female rats by attenuating oxidative stress, inflammation and apoptosis. Chem Biol Interact. 2021;338:109402. 33587916 10.1016/j.cbi.2021.109402

[7] Zhang CR Zhu WN Tao W. Moxibustion against cyclophosphamide-induced premature ovarian failure in rats through inhibiting NLRP3-/Caspase-1-/GSDMD-dependent pyroptosis. Evid Based Complement Alternat Med. 2021;2021:8874757. 33613687 10.1155/2021/8874757 PMC7878072

[8] Deng T He J Yao Q. Human umbilical cord mesenchymal stem cells improve ovarian function in chemotherapy-induced premature ovarian failure mice through inhibiting apoptosis and inflammation via a paracrine mechanism. Reprod Sci. 2021;28:1718–32. 33751459 10.1007/s43032-021-00499-1

[9] Alharbi H Alshehri AS Ahmad M. Promising anti-cervical carcinoma and inflammatory agent, resveratrol targets poly (ADP-ribose) polymerase 1 (PARP-1) induced premature ovarian failure with a potent enzymatic modulatory activity. J Reprod Immunol. 2021;144:103272. 33465522 10.1016/j.jri.2021.103272

[10] Zhu X Liu J Pan H. Thymopentin treatment of murine premature ovarian failure via attenuation of immune cell activity and promotion of the BMP4/Smad9 signalling pathway. Int J Med Sci. 2021;18:3544–55. 34522181 10.7150/ijms.61975 PMC8436114

[11] Lu X Cui J Cui L. The effects of human umbilical cord-derived mesenchymal stem cell transplantation on endometrial receptivity are associated with Th1/Th2 balance change and uNK cell expression of uterine in autoimmune premature ovarian failure mice. Stem Cell Res Ther. 2019;10:214. 31331391 10.1186/s13287-019-1313-y PMC6647296

[12] Yin N Wang Y Lu X. hPMSC transplantation restoring ovarian function in premature ovarian failure mice is associated with change of Th17/Tc17 and Th17/Treg cell ratios through the PI3K/Akt signal pathway. Stem Cell Res Ther. 2018;9:37. 29444704 10.1186/s13287-018-0772-x PMC5813427

[13] Andersson-Sjöland A Karlsson JC Rydell-Törmänen K. ROS-induced endothelial stress contributes to pulmonary fibrosis through pericytes and Wnt signaling. Lab Invest. 2016;96:206–17. 26367492 10.1038/labinvest.2015.100

[14] Sha C Chen L Lin L. TRDMT1 participates in the DNA damage repair of granulosa cells in premature ovarian failure. Aging (Albany NY). 2021;13:15193–213. 34100772 10.18632/aging.203080 PMC8221345

[15] Zhang M Yu X Li D. Nrf2 signaling pathway mediates the protective effects of daphnetin against D-galactose induced-premature ovarian failure. Front Pharmacol. 2022;13:810524. 35153783 10.3389/fphar.2022.810524 PMC8832979

[16] Zheng S Ma M Chen Y. Effects of quercetin on ovarian function and regulation of the ovarian PI3K/Akt/FoxO3a signalling pathway and oxidative stress in a rat model of cyclophosphamide-induced premature ovarian failure. Basic Clin Pharmacol Toxicol. 2022;130:240–53. 34841658 10.1111/bcpt.13696

[17] Samarakoon R Overstreet JM Higgins PJ. TGF-β signaling in tissue fibrosis: redox controls, target genes and therapeutic opportunities. Cell Signal. 2013;25:264–8. 23063463 10.1016/j.cellsig.2012.10.003 PMC3508263

[18] Li F Zhang A Shi Y. 1α,25-Dihydroxyvitamin D3 prevents the differentiation of human lung fibroblasts via microRNA-27b targeting the vitamin D receptor. Int J Mol Med. 2015;36:967–74. 26311239 10.3892/ijmm.2015.2318 PMC4564074

[19] Zhou F Shi LB Zhang SY. Ovarian fibrosis: a phenomenon of concern. Chin Med J (Engl). 2017;130:365–71. 28139522 10.4103/0366-6999.198931 PMC5308021

[20] Yamchi NN Rahbarghazi R Bedate AM. Menstrual blood CD146(+) mesenchymal stem cells reduced fibrosis rate in the rat model of premature ovarian failure. Cell Biochem Funct. 2021;39:998–1008. 34477225 10.1002/cbf.3669

[21] Carlberg C. Vitamin D signaling in the context of innate immunity: focus on human monocytes. Front Immunol. 2019;10:2211. 31572402 10.3389/fimmu.2019.02211 PMC6753645

[22] Zhou L Wang J Li J. 1,25-Dihydroxyvitamin D3 Ameliorates collagen-induced arthritis via suppression of Th17 cells through miR-124 mediated inhibition of IL-6 signaling. Front Immunol. 2019;10:178. 30792721 10.3389/fimmu.2019.00178 PMC6374300

[23] El-Sharkawy A Malki A. Vitamin D signaling in inflammation and cancer: molecular mechanisms and therapeutic implications. Molecules. 2020;25:3219. 32679655 10.3390/molecules25143219 PMC7397283

[24] Dong B Zhou Y Wang W. Vitamin D receptor activation in liver macrophages ameliorates hepatic inflammation, steatosis, and insulin resistance in mice. Hepatology. 2020;71:1559–74. 31506976 10.1002/hep.30937

[25] Yu X Liu B Zhang N. Immune response: a missed opportunity between vitamin D and radiotherapy. Front Cell Dev Biol. 2021;9:646981. 33928081 10.3389/fcell.2021.646981 PMC8076745

[26] Ma JG Wu GJ Xiao HL. Vitamin D has an effect on airway inflammation and Th17/Treg balance in asthmatic mice. Kaohsiung J Med Sci. 2021;37:1113–21. 34460994 10.1002/kjm2.12441 PMC11896363

[27] Gibbs DC Fedirko V Baron JA. Inflammation modulation by vitamin D and calcium in the morphologically normal colorectal mucosa of patients with colorectal adenoma in a clinical trial. Cancer Prev Res (Phila). 2021;14:65–76. 32917645 10.1158/1940-6207.CAPR-20-0140 PMC7947029

[28] Hajiluian G Abbasalizad Farhangi M Nameni G. Oxidative stress-induced cognitive impairment in obesity can be reversed by vitamin D administration in rats. Nutr Neurosci. 2018;21:744–52. 28683595 10.1080/1028415X.2017.1348436

[29] Berridge MJ. Vitamin D, reactive oxygen species and calcium signalling in ageing and disease. Philos Trans R Soc Lond B Biol Sci. 2016;371:20150434. 27377727 10.1098/rstb.2015.0434 PMC4938033

[30] Ricca C Aillon A Bergandi L. Vitamin D receptor is necessary for mitochondrial function and cell health. Int J Mol Sci. 2018;19:1672. 29874855 10.3390/ijms19061672 PMC6032156

[31] Adam-Bonci TI Bonci EA Pârvu AE. Vitamin D supplementation: oxidative stress modulation in a mouse model of ovalbumin-induced acute asthmatic airway inflammation. Int J Mol Sci. 2021;22:7089. 34209324 10.3390/ijms22137089 PMC8268667

[32] Kaplan S Türk A Aydin H. Vitamin D improves oxidative stress and histopathological damage in rat ovaries caused by hyperthyroidism. J Obstet Gynaecol Res. 2021;47:3551–60. 34291533 10.1111/jog.14948

[33] Kim J Kang W Kang SH. Proline-rich tyrosine kinase 2 mediates transforming growth factor-beta-induced hepatic stellate cell activation and liver fibrosis. Sci Rep. 2020;10:21018. 33273492 10.1038/s41598-020-78056-0 PMC7713048

[34] Chang J Nie H Ge X. Vitamin D suppresses bleomycin-induced pulmonary fibrosis by targeting the local renin-angiotensin system in the lung. Sci Rep. 2021;11:16525. 34400742 10.1038/s41598-021-96152-7 PMC8367953

[35] Sun S Xu M Zhuang P. Effect and mechanism of vitamin D activation disorder on liver fibrosis in biliary atresia. Sci Rep. 2021;11:19883. 34615940 10.1038/s41598-021-99158-3 PMC8494743

[36] Sun H Shi Y Shang Y. MicroRNA-378d inhibits Glut4 by targeting Rsbn1 in vitamin D deficient ovarian granulosa cells. Mol Med Rep. 2021;23:369. 33760197 10.3892/mmr.2021.12008 PMC7985995

[37] Wan T Sun H Mao Z. Vitamin D deficiency inhibits microRNA-196b-5p which regulates ovarian granulosa cell hormone synthesis, proliferation, and apoptosis by targeting RDX and LRRC17. Ann Transl Med. 2021;9:1775. 35071469 10.21037/atm-21-6081 PMC8756257

[38] Barrera-Vargas A Gómez-Martín D Carmona-Rivera C. Differential ubiquitination in NETs regulates macrophage responses in systemic lupus erythematosus. Ann Rheum Dis. 2018;77:944–50. 29588275 10.1136/annrheumdis-2017-212617 PMC6560641

[39] Teijeira L Garasa S Gato M. CXCR1 and CXCR2 chemokine receptor agonists produced by tumors induce neutrophil extracellular traps that interfere with immune cytotoxicity. Immunity. 2020;52:856–71.e8. 32289253 10.1016/j.immuni.2020.03.001

[40] Döring Y Libby P Soehnlein O. Neutrophil extracellular traps participate in cardiovascular diseases: recent experimental and clinical insights. Circ Res. 2020;126:1228–41. 32324499 10.1161/CIRCRESAHA.120.315931 PMC7185047

[41] Zucoloto AZ Jenne CN. Platelet-neutrophil interplay: insights into Neutrophil Extracellular Trap (NET)-driven coagulation in infection. Front Cardiovasc Med. 2019;6:85. 31281822 10.3389/fcvm.2019.00085 PMC6595231

[42] Zhang H Qiu SL Tang QY. Erythromycin suppresses neutrophil extracellular traps in smoking-related chronic pulmonary inflammation. Cell Death Dis. 2019;10:678. 31515489 10.1038/s41419-019-1909-2 PMC6742640

[43] Linhares-Lacerda L Temerozo JR Ribeiro-Alves M. Neutrophil extracellular trap-enriched supernatants carry microRNAs able to modulate TNF-α production by macrophages. Sci Rep. 2020;10:2715. 32066757 10.1038/s41598-020-59486-2 PMC7026108

[44] Zhu B Zhang X Sun S. NF-κB and neutrophil extracellular traps cooperate to promote breast cancer progression and metastasis. Exp Cell Res. 2021;405:112707. 34153301 10.1016/j.yexcr.2021.112707

[45] Grégoire M Uhel F Lesouhaitier M. Impaired efferocytosis and neutrophil extracellular trap clearance by macrophages in ARDS. Eur Respir J. 2018;52:1702590. 29946009 10.1183/13993003.02590-2017

[46] Wang J Liu Z Han Z. Fumonisin B1 triggers the formation of bovine neutrophil extracellular traps. Toxicol Lett. 2020;332:140–5. 32659472 10.1016/j.toxlet.2020.07.006

[47] Ye S Li S Ma Y. Curcumin hinders PBDE-47-induced neutrophil extracellular traps release via Nrf2-associated ROS inhibition. Ecotoxicol Environ Saf. 2021;225:112779. 34530259 10.1016/j.ecoenv.2021.112779

[48] Jiang L Gao X Xu J. Alumina nanoparticles-induced heterophil extracellular traps exacerbate liver injury by regulating oxidative stress and inflammation in chickens. J Inorg Biochem. 2022;229:111725. 35063926 10.1016/j.jinorgbio.2022.111725

[49] Gaul DS Weber J van Tits LJ. Loss of Sirt3 accelerates arterial thrombosis by increasing formation of neutrophil extracellular traps and plasma tissue factor activity. Cardiovasc Res. 2018;114:1178–88. 29444200 10.1093/cvr/cvy036 PMC6014146

[50] Edwards NJ Hwang C Marini S. The role of neutrophil extracellular traps and TLR signaling in skeletal muscle ischemia reperfusion injury. FASEB J. 2020;34:15753–70. 33089917 10.1096/fj.202000994RR PMC8054227

[51] Zenlander R Havervall S Magnusson M. Neutrophil extracellular traps in patients with liver cirrhosis and hepatocellular carcinoma. Sci Rep. 2021;11:18025. 34504150 10.1038/s41598-021-97233-3 PMC8429678

[52] Aldabbous L Abdul-Salam V McKinnon T. Neutrophil extracellular traps promote angiogenesis: evidence from vascular pathology in pulmonary hypertension. Arterioscler Thromb Vasc Biol. 2016;36:2078–87. 27470511 10.1161/ATVBAHA.116.307634

[53] Negreros M Flores-Suárez LF. A proposed role of neutrophil extracellular traps and their interplay with fibroblasts in ANCA-associated vasculitis lung fibrosis. Autoimmun Rev. 2021;20:102781. 33609801 10.1016/j.autrev.2021.102781

[54] Suzuki M Ikari J Anazawa R. PAD4 deficiency improves bleomycin-induced neutrophil extracellular traps and fibrosis in mouse lung. Am J Respir Cell Mol Biol. 2020;63:806–18. 32915635 10.1165/rcmb.2019-0433OC

[55] Chen C Weng H Zhang X. Low-dose vitamin D protects hyperoxia-induced bronchopulmonary dysplasia by inhibiting neutrophil extracellular traps. Front Pediatr. 2020;8:335. 32719755 10.3389/fped.2020.00335 PMC7347751

[56] Elfayomy AK Almasry SM El-Tarhouny SA. Human umbilical cord blood-mesenchymal stem cells transplantation renovates the ovarian surface epithelium in a rat model of premature ovarian failure: possible direct and indirect effects. Tissue Cell. 2016;48:370–82. 27233913 10.1016/j.tice.2016.05.001

